# Exosome-Seeded Cryogel Scaffolds for Extracellular Matrix Regeneration in the Repair of Articular Cartilage Defects: An In Vitro and In Vivo Rabbit Model Study

**DOI:** 10.3390/polym17070975

**Published:** 2025-04-03

**Authors:** Daniel Yang, Joseph Yang, Shwu-Jen Chang, Jhe-Lun Hu, Yong-Ji Chen, Shan-Wei Yang

**Affiliations:** 1 Laboratory of Regenerative Medicine and Biosensors, I-Shou University, Kaohsiung City 824005, Taiwan; danielyang0930@gmail.com (D.Y.); josephyang0218@gmail.com (J.Y.); sjchang@isu.edu.tw (S.-J.C.); alanhu0812@gmail.com (J.-L.H.); ygchen2021@gmail.com (Y.-J.C.); 2 Cambridge International Programme, St. Dominic Catholic High School, Kaohsiung City 802306, Taiwan; 3 Department of Biomedical Engineering, I-Shou University, Kaohsiung City 824005, Taiwan; 4 Department of Orthopedics, Kaohsiung Veterans General Hospital, Kaohsiung City 813414, Taiwan; 5 School of Nursing, Fooyin University, Kaohsiung City 831301, Taiwan; 6 Department of Leisure and Sports Management, Cheng Shiu University, Kaohsiung City 833301, Taiwan

**Keywords:** cryogel, exosome, extracellular matrix, hyaluronic acid, articular cartilage repair

## Abstract

Traumatic or degenerative defects of articular cartilage impair joint function, and the treatment of articular cartilage damage remains a challenge. By mimicking the cartilage extracellular matrix (ECM), exosome-seeded cryogels may enhance cell proliferation and chondral repair. ECM-based cryogels were cryopolymerized with gelatin, chondroitin sulfate, and various concentrations (0%, 0.3%, 0.5%, and 1%) of hyaluronic acid (HA), and their water content, swelling ratio, porosity, mechanical properties, and effects on cell viability were evaluated. The regenerative effects of bone marrow-derived mesenchymal stem cell (BM-MSC)-derived exosome (at a concentration of 10^6^ particles/mL)-seeded 0.3% HA cryogels were assessed in vitro and in surgically induced male New Zealand rabbit cartilage defects in vivo. The water content, swelling ratio, and porosity of the cryogels significantly (*p* < 0.05) increased and the Young’s modulus values of the cryogels decreased with increasing HA concentrations. MTT assays revealed that the developed biomaterials had no cytotoxic effects. The optimal cryogel composition was 0.3% HA, and the resulting cryogel had favorable properties and suitable mechanical strength. Exosomes alone and exosome-seeded cryogels promoted chondrocyte proliferation (with cell optical densities that were 58% and 51% greater than that of the control). The cryogel alone and the exosome-seeded cryogel facilitated ECM deposition and sulfated glycosaminoglycan synthesis. Although we observed cartilage repair via Alcian blue staining with both the cryogel alone and the exosome-seeded cryogel, the layered arrangement of the chondrocytes was superior to that of the control chondrocytes when exosome-seeded cryogels were used. This study revealed the potential value of using BM-MSC-derived exosome-seeded ECM-based cryogels for cartilage tissue engineering to treat cartilage injury.

## 1. Introduction

Articular cartilage, which is hyaline cartilage on the articular surfaces of diarthrodial joints, is a thin layer of specialized connective tissue that is bathed in synovial fluid, providing a smooth and lubricating surface for articulation and for distributing loads exerted on the synovial joints. Articular cartilage is composed of three different zones, which are categorized according to the orientation of type II collagen and the shape of chondrocytes: the superficial zone, the intermediate zone, and the deep zone. The tidemark separates the deep zone from the calcified zone, which anchors the collagen fibers of the deep zone to the subchondral bone [1]. The extracellular matrix (ECM) of articular cartilage consists of water, collagen, and proteoglycans. Specifically, type II collagen accounts for 90–95% of the total collagen content, stabilizing the ECM and providing shear and tensile properties to resist the forces originating from articulation [2]. Aggrecan, the most abundant proteoglycan, contains sulfated glycosaminoglycans (sGAGs), such as chondroitin sulfate (CS) and keratin sulfate (KS), and aggregates with hyaluronic acid (HA) to provide compressive strength and to attract water molecules.

Cartilage defects may be traumatic, resulting from acute mechanical overload, or degenerative, resulting from wear and tear due to advanced age or obesity. Articular cartilage destruction may be attributable to the chondrocyte apoptosis induced by friction on a rough cartilage surface, particularly in the superficial zone [3]. Defects not only disrupt normal joint function but also lead to pain and inflammation, limiting mobility. As the articular cartilage is avascular and has limited self-healing potential, it does not regenerate after chondral injuries that are limited to the thin layer, impacting the smooth surface needed for the flow of synovial fluid. The currently available clinical strategies for treating damaged cartilage include physical rehabilitation, medications, and injection of lubrication substances to achieve symptomatic control [4]. However, these treatments are not able to achieve disease modification with cartilage repair, and the healing and repair of injured cartilage remains a challenging clinical problem [5]. The currently available surgical treatment options for cartilage repair include microfracture, which involves the induction of mesenchymal progenitor cells from the bone marrow to synthesize fibrocartilage and has drawbacks such as inferior quality and longevity [6]; osteochondral grafting (also known as mosaicplasty), which is reported to be effective longitudinally but has limitations such as limited donor tissue availability [7]; and autologous chondrocyte implantation, which requires a two-step surgical procedure [8]. Further developments in cartilage tissue engineering may play a role in the treatment of articular cartilage injuries.

Cryogels are gel matrices with interconnected supermacropores that are formed from monomers or polymeric molecules and undergo cryogelation at subzero temperatures [9]. They are formed through a series of processes: ice crystals act as porogen and induce pore formation, cross-linking, and cryopolymerization; an interconnecting porous structure is created after the ice crystals are thawed. The three-dimensional hydrophilic and electrically charged polymer network of cryogels acts as a scaffold to allow tissue regeneration and cell proliferation, with cells migrating into the structure. The structure of cryogel is chemically and mechanically stable, which allows biological nanomolecules such as organelles to be bound and immobilized [10]. The macroporosity of cryogels allows the influx of nutrients and solutes into the structure, enables the transport of cellular waste out of the cells, and enhances ECM deposition by the cells [11].

Compared with hydrogels, another type of ECM-mimetic material, cryogels differ in their characteristics and synthesis mechanism. When cryogels are dehydrated, lyophilized, and thawed, the interconnected macropores allow more solution to be absorbed into the cryogels, resulting in a swelling ratio that is greater than that of freeze-dried hydrogels [12]. This result occurs because the thin walls and solvent channels enable the rapid transport of solvent molecules over short distances [13]—the solvent moves through the walls in the cryogel via convection around the pores, in contrast to the diffusion that occurs in hydrogels [14]. Additionally, cryogels have shown promise in tissue engineering due to their superior stability and mechanical robustness compared with hydrogels [15]. The high rigidity of cryogel polymers allows the structure to be maintained even when the cryogel is dried [16]. Considering this context, we selected cryogels rather than conventional hydrogels as our 3-dimensional (3D) scaffold for carrying exosomes.

We synthesized ECM-based cryogels from gelatin, HA, and CS. Gelatin is a natural polypeptide that is soluble in water and nontoxic to humans and is derived from the hydrolysis of the triple-helix structure of collagen; it contains many arginine–glycine–aspartate (RGD) tripeptides, which can be used as cellular binding sites for cell adhesion and target sequences for matrix metalloproteinases, making gelatin easily biodegradable and initiating minimal immune response [17]. Furthermore, gelatin is an FDA-approved material because of its biodegradability and biocompatibility. HA is a naturally occurring high-molecular-weight polysaccharide with essential biological functions in joint synovial fluid, such as lubricating and regulating protein expression in chondrocytes, transporting nutrients and metabolites, and stabilizing the structure of collagen [18]. In addition, HA binds to chondrocytes and other molecules or receptors in the ECM, such as aggrecan and the cell surface adhesion glycoprotein CD44, stabilizing the ECM structure and regulating cellular signal transduction to mediate water transport and proteoglycan assembly [19]. Additionally, the negative charges of the polymer chain allow the cryogel to completely unfold in water due to electrostatic repulsion; it then occupies considerable space and is capable of taking up a large amount of water [20]. CS is an sGAG that is usually attached to proteins as part of a proteoglycan and contributes to the tensile strength of cartilage by strengthening the hydrogen and electrostatic bonding organization of collagen fibrils [21]. CS is often used in combination with other ingredients, such as HA, for the treatment of chondral defects as it slows the breakdown of cartilage by inhibiting lipoxidation and protein oxidation reactions and is one of the main building blocks of cartilage.

Exosomes are extracellular vesicles that are secreted by the endosomal compartment of eukaryotic cells via exocytosis and carry substances such as nucleic acids, proteins, lipids, and metabolites. It is possible to use exosomes to target complex intracellular pathways to achieve therapeutic effects, including in the context of cancer therapy, as exosomes can achieve targeted drug delivery with minimal immune responses [22]. This cell-free therapeutic approach has several advantages over previously employed clinical strategies, such as intravenous fusion of mesenchymal stem cells (MSCs), which has been associated with toxicity and tumorigenesis [23]. Exosomes are among the main secretory products of MSCs, and, therefore, the effects of MSC exosomes resemble the effects of their parent MSCs; exosomes from bone marrow-derived mesenchymal stem cells (BM-MSCs) have been shown to restore the expression of aggrecan and collagen type II while inhibiting the expression of catabolic and inflammatory markers in osteoarthritis-like chondrocytes [24]. Exosomes have the potential to remodel the ECM and transmit signaling molecules by transporting molecules or accumulating specific cellular components [25]. It may be possible to treat traumatic chondral injuries with exosomes by inhibiting the inflammatory cascade and restoring the balance between ECM catabolic and anabolic pathways [26]. In addition, BM-MSC-derived exosomes have been shown to promote synovial macrophage polarization, inhibiting proinflammatory macrophage generation and promoting anti-inflammatory macrophage production [27]. Furthermore, specific components of exosomes have been shown to stimulate the chondrogenic differentiation of BM-MSCs into chondrocytes [28] and improve cartilage regeneration through increased chondrocyte proliferation.

An in vitro study showed the potential of using a chitosan–gelatin–chondroitin (CGC) cryogel extract for the delivery of exosomes to promote chondrocyte proliferation and migration [29]. However, no previous in vivo study has evaluated the effectiveness of exosome-seeded cryogels with the addition of HA for articular cartilage defect repair. To evaluate the potential use of BM-MSC-derived exosome-seeded ECM-based cryogels for the repair of articular cartilage defects, we fabricated gelatin–CS–HA cryopolymerized cryogels for cartilage tissue engineering. The water content, swelling ratio, porosity, pore size, mechanical stress behavior, and cell viability of the cryogels were evaluated to determine the optimum HA concentration. The optimum BM-MSC-derived exosome concentration was determined concerning cell viability. We also conducted chondrocyte proliferation assays, biochemical analyses, and in vivo implantation, with histological staining analyses, into a rabbit experimental model.

## 2. Materials and Methods

### 2.1. Synthesis of the Extracellular Matrix-Based Cryogel

First, an amount of 0 g (0% *w*/*v* condition), 0.03 g (0.3% *w*/*v* condition), 0.05 g (0.5% *w*/*v* condition), or 0.1 g (1% *w*/*v* condition) of HA (MW: 1,000,000 Da; Kinkasei ChemoLab, Taichung City, Taiwan) was dissolved in 9 mL of double-distilled water (ddH_2_O) containing 4 g (40% *w*/*v*) of gelatin from type B bovine skin (225 g Bloom, MW: 50,000 Da; Sigma-Aldrich, St. Louis, MO, USA) and 0.1 g (1% *w*/*v*) of CS sodium salt from bovine cartilage (lyophilized powder; Sigma-Aldrich, St. Louis, MO, USA) and heated to 70 °C. Furthermore, 0.005 g (0.05% *w*/*v*) of the gelling agent 1-(3-dimethylaminopropyl)-3-ethylcarbodiimide hydrochloride (98+%; Thermo Fisher Scientific, Waltham, MA, USA) was dissolved in 1 mL of ddH_2_O while stirring, resulting in a final volume of 10 mL. The solution was poured into a 5 cm^3^ syringe mold, which was then sealed with Parafilm^®^ M and oscillated in an ultrasonic cleaner for 30 s. The solution, along with the sealed mold, was frozen overnight at −20 °C in absolute ethanol for the cross-linking reaction.

The materials were cut into 3 mm thick discs with a diameter of approximately 10 mm and dehydrated serially with 25%, 50%, 75%, and absolute ethanol for 3 min each. The dehydrated sample discs were impregnated with absolute ethanol and frozen at −20 °C for 12 h. Then, the sample discs were laid out on a Petri dish, the ethanol was allowed to evaporate for 1 to 2 h, and the samples were then frozen at −20 °C for 8 to 12 h. Finally, the sample discs were lyophilized at −90 °C for 12 to 24 h to obtain the ECM-based cryogels.

### 2.2. Water Content Analysis

The dry mass (M_dry_) of each cryogel disc sample was measured before it was impregnated with ddH_2_O for 1 h. The wet mass (M_wet_) of the cryogels was calculated by subtracting the remaining mass of ddH_2_O (M_remaining_) on a top pan scale with a precision of 0.0001 g from the wicked mass (M_wicked_), with the extraneous water removed from the surface with Kimwipes^™^ (M_wet_ = M_wicked_ − M_remaining_). The water content was calculated as a percentage with the formula (M_wet_ − M_dry_)/M_wet_ × 100%.

### 2.3. Swelling Ratio Analysis

The dry masses (M_dry_) of the cryogel disc samples was measured before they were impregnated with ddH_2_O, and the data were measured at 5 min, 10 min, 15 min, 30 min, 1 h, 2 h, 3 h, 6 h, 12 h, 24 h, 36 h, 48 h, and 72 h after swelling. The wet mass (M_wet_) of the cryogels was calculated by subtracting the remaining mass of ddH_2_O (M_remaining_) on a top pan scale with a precision of 0.0001 g from the wicked mass (M_wicked_), with the extraneous water removed from the surface with Kimwipes^TM^ (M_wet_ = M_wicked_ − M_remaining_). The swelling ratio was calculated as a percentage with the formula (M_wet_ − M_dry_)/M_dry_ × 100%.

### 2.4. Porosity Analysis

The dry mass (M_dry_) and the volume (V) of the cryogel disc samples, which was calculated from the thickness and diameter using a caliper with a precision of 0.05 mm, were measured before they were impregnated with absolute ethanol for 10 min. Cryogels before swelling and after swelling in ddH_2_O for 24 h were used, with the swollen cryogels lyophilized at −90 °C for 12 to 24 h. The wet mass (M_wet_) of the cryogels was calculated by subtracting the remaining mass of ethanol (M_remaining_) on a top pan scale with a precision of 0.0001 g from the wicked mass (M_wicked_), with the extraneous ethanol removed from the surface with Kimwipes^™^ (M_wet_ = M_wicked_ − M_remaining_). The porosity was calculated as a percentage by using the formula [(M_wet_ − M_dry_)/ρ_ethanol_]/V × 100%, with the density of ethanol (ρ_ethanol_) equal to 0.789 g/cm^3^.

### 2.5. Scanning Electron Microscopy Analysis

The cryogels were lyophilized and vacuum sputter-coated with gold (Fine Coat Ion Sputter JFC 1100, JEOL, Tokyo, Japan) to increase the conductivity of the materials. An analytical field emission scanning electron microscope (SEM) (Zeiss Auriga FIB-SEM) was used to image the cryogels at 10 kV to evaluate the pore size, surface cross-sectional structure, and compactness of the 3D scaffold. The pore size of the swollen cryogel after 24 h was measured.

### 2.6. Mechanical Strength Analysis

The compressive Young’s modulus of the swollen cryogels at 6 h was measured using a universal material test machine (Tinius Olsen, Kongsberg, Norway) to perform a uniaxial compression evaluation with a strain rate of 0.5 mm/min. The strength of the force in Newtons and the change in thickness in mm were recorded up to the fracture point. All the data were converted into stress in MPa and strain as a percentage and Young’s modulus was calculated from the linear region of the stress–strain graph.

### 2.7. Cell Viability Analysis

An MTT (3-(4,5-dimethylthiazol-2-yl)-2,5-diphenyltetrazolium bromide) assay was used to evaluate the cytotoxicity of the material and its effects on cell viability compared with that of untreated cells. A total of 10^5^ L929 mouse fibroblasts obtained from the Bioresource Collection and Research Center (BCRC), Hsinchu, Taiwan, were seeded onto cryogels in a 24-well plate, cultured in 0.5 mL of high-glucose Dulbecco’s modified Eagle’s medium supplemented with 10% *w*/*v* fetal bovine serum and 1% w/v penicillin–streptomycin, and filtered through a 0.22 μm sterilized filter after 24 h at 37 °C and 5% CO_2_ for initial testing. The cells were cultured without cryogel or with 0%, 0.3%, 0.5%, or 1% HA cryogel for 48 h, and the extract in the wells was removed by washing with 1X phosphate-buffered saline (PBS).

The cells were then incubated with serum-free medium containing 0.5 mg/mL MTT for 3 h before centrifugation at 12,000× *g* rpm for 5 min, and the formazan products were completely dissolved in 0.25 mL dimethyl sulfoxide by vortexing. The samples were transferred to a new plate and evaluated at a wavelength of 570 nm using an ELISA microplate photometer (SPECTROstar Nano, BMG Labtech, Ortenberg, Germany) to determine the light absorbance value.

To determine the optimal concentration of BM-MSC-derived exosomes, 3-week-old New Zealand white rabbits were sacrificed by cardiac injection of saturated potassium chloride after inhalational anesthesia with isoflurane to obtain the articular cartilage tissue using a surgical blade that had been washed three times with PBS. The cartilage fragments were soaked in serum-free medium containing 2 mg/mL proteinase for 2 h, followed by dissociation in serum-free F12 medium containing 2 mg/mL collagenase II for 3 h. The chondrocytes were collected after centrifugation at 1500× *g* rpm for 10 min to remove the upper layer of the supernatant, with the isolated cells cultured in F12 culture medium containing 2 mg/mL proteinase, 10% *w*/*v* bovine serum, and antibiotics and incubated at 37 °C and 5% CO_2_ in 75T cell culture flasks. Chondrocytes treated with exosomes at concentrations of 10^3^, 10^4^, 10^5^, 10^6^, and 10^7^ particles/mL were cultured for 24, 48, 72, and 168 h, and MTT assays were carried out using the procedure described above.

### 2.8. Bone Marrow-Derived Mesenchymal Stem Cell-Derived Exosome Analysis

BM-MSC-derived exosome samples were obtained from Yia Jia Biotechnology Co., Ltd. (YJ, New Taipei City, Taiwan). Umbilical cord tissues were obtained from full-term deliveries of healthy donors who had provided written informed consent prior to collection. The study protocol was reviewed and approved by the Institutional Review Board of Fu Jen Catholic University (Institutional Review Board Approval No. C111157) and was conducted in accordance with the principles of the Declaration of Helsinki. Donor peripheral blood samples were screened and confirmed negative for hepatitis B virus, human immunodeficiency viruses 1 and 2, hepatitis C virus, and both syphilis antigens and antibodies. The isolation and expansion of MSCs were performed in a Good Manufacturing Practice-compliant cell processing facility (EZCPi ONE, YJ Biotechnology, New Taipei City, Taiwan), certified by the Taiwan Food and Drug Administration under the Good Tissue Practice guidelines [30].

Exosomes were adsorbed onto aldehyde/sulfate latex beads (Thermo Fisher Scientific, Waltham, MA, USA) at a concentration of 4% *w*/*v* and a size of 4 µm for 1 h, with the same number of beads and exosomes, to ensure their surface marker. Then, 100 mM glycine was added to stop the reaction and the mixture was washed with 1 mL of PBS. The exosomes were cultured with monoclonal anti-CD63 antibodies produced in mice at room temperature for 1 h, after which they were stained with fluorescein isothiocyanate-conjugated secondary antibodies for 1 h at room temperature. After being washed with PBS, the samples were resuspended in 1X PBS and analyzed using a flow cytometer (ImageStream^®X^ Mark II, Amnis, Vaduz, Liechtenstein) to determine the concentration of exosomes.

Nanoparticle tracking analysis (NTA) (NanoSight LM10-HS, Malvern Panalytical, Malvern, UK) was carried out with the purified exosomes diluted in PBS at a ratio of 1:1000. The concentration and size distribution of the exosomes were determined by recording the frequency shifts of light from the coherent light source due to the movement of particles scattering the light. A transmission electron microscope (JEM-1400, JEOL, Tokyo, Japan) was used to obtain micrographs by glow discharging the carbon-coated copper transmission electron microscopy (TEM) grid, pipetting the exosomes suspended in PBS on the copper grid, and then subjecting the sample to negative staining after removing the excess solvent.

### 2.9. Chondrocyte Proliferation Analysis

MTT assays were carried out with chondrocytes extracted using the method described above, which were treated with either the cryogel alone, exosomes alone, or exosome-seeded cryogel and cultured for 24, 48, 72, and 168 h.

The EZViableTM Calcein AM Cell Viability Assay Kit (BioVision Research, Milpitas, CA, USA) was used according to the manufacturer’s protocol to confirm the viability of the chondrocytes that migrated into the scaffold. In brief, chondrocytes were treated with either the cryogel alone, exosomes alone, or exosome-seeded cryogel and cultured for 24, 48, 72, and 168 h. Cultures were stained with 1 mM dye and viewed under a fluorescence microscope, with an excitation wavelength of 488 nm and an emission wavelength of 515 nm. The intensity of the Calcein AM staining in each sample was measured using ImageJ software (Version 1.53t, National Institutes of Health, Bethesda, MD, USA).

### 2.10. Biochemical Analysis

For the Alcian blue analysis, chondrocytes were treated with either the cryogel alone, exosomes alone, or exosome-seeded cryogel and cultured for 24, 48, 72, and 168 h. For the cryogel condition, cryogel was coated onto the surface of the culture wells, and chondrocytes were directly seeded on top of the cryogel surface. Afterward, the chondrocytes were incubated overnight in a 1% *w*/*v* Alcian blue solution. After three rinses with PBS, the samples were observed and imaged with an optical microscope. The intensity of the Alcian blue staining in each sample was measured using ImageJ software.

The sGAG content was evaluated using a Blyscan Assay Kit (Biocolor, Belfast, Northern Ireland) according to the manufacturer’s instructions. Chondrocytes were treated with either the cryogel alone, exosomes alone, or exosome-seeded cryogel and cultured for 24, 48, 72, and 168 h. In brief, the culture medium was collected and mixed with 1 mL of dye for 30 min, and the samples were mixed with 1 mL of dissociation reagent for 20 min after centrifugation at 12,000× *g* rpm for 10 min to remove the supernatant. The absorbance at 650 nm was measured using a microplate reader, and the sGAG content was calculated from the standard curve.

### 2.11. In Vivo Implantation Analysis

**Figure 1:**
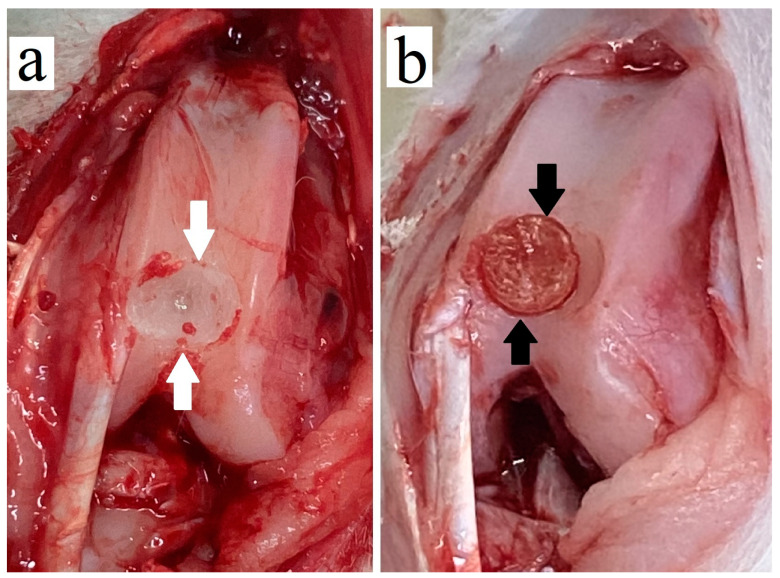
In vivo implantation of cryogel. (**a**) Surgically induced chondral defects (white arrows) in the femoral articular cartilage of the rabbit knee joint; (**b**) implantation of the extracellular matrix (ECM)-based cryogel (black arrows) over the defect.

Chondral defects 5 mm in diameter and 2 mm deep were generated in the femoral articular cartilage of knee joints without damaging the subchondral bone in 12-week-old healthy male New Zealand rabbits with no previous surgical experience and an average weight of 2.9 ± 0.2 kg. The defects were generated to model articular cartilage damage, and this was achieved via surgical drilling in a germ-free room after inhalational anesthesia with isoflurane (Figure 1a). After sterilization via ultraviolet radiation for 1 to 2 h, the cryogels were implanted into the chondral defects (Figure 1b), and the animals were sacrificed four weeks after surgery via cardiac injection of saturated KCl solution under anesthesia.

All of the experimental limbs were divided randomly into three groups: those treated with the exosome-seeded cryogel (*n* = 2), those treated with the cryogel without exosomes (*n* = 2), and those that received no treatment as the control group (*n* = 2). The cryogels were impregnated with BM-MSC-derived exosomes for 24 h at 4° C and then frozen at −20 °C for 1 to 2 h before being lyophilized at −90 °C for 12 to 24 h to obtain the exosome-seeded cryogels.

The three rabbits were caged individually and treated in a randomized order. The sample size was designed in accordance with the 3Rs Principle of Animal Research (replacement, reduction, and refinement) to minimize the number of animals used in the preliminary study, and all animals were included in the analysis. All animals had free access to food and water. The animals received Neomycin Ointment “N.Y.” (5 mg/g; Genuine Chemical Pharmaceutical, Chungli, Taiwan) once daily for antibiotic prophylaxis and Buprenorphine (0.03 mg/kg; Bayer, Zürich, Switzerland) twice daily for analgesia over one week. Animal welfare was continuously monitored daily and evaluated based on the condition of the surgical wound, food and water intake, body weight, and movement behavior.

### 2.12. Histological Analysis

Sagittal sections of the operated cartilage were collected and fixed in 10% *w*/*v* formalin for 24 h, decalcified with Gooding and Stewart’s fluid (equal volumes of 10% *w*/*v* formalin and 10% *w*/*v* formic acid solution), and embedded in paraffin. Hematoxylin and eosin (H&E) staining and Alcian blue staining were employed to observe the damaged cartilage layers and to evaluate the impact of the ECM-based BM-MSC-derived exosome-seeded cryogel on cartilage repair. For the Alcian blue staining, the chondrocytes were incubated overnight in a 1% *w*/*v* Alcian blue solution. The sections were imaged using an optical microscope after three washes with PBS. This study was conducted using a single-blind design, where the evaluator was unaware of the models’ group. The sections were not only evaluated based on the completeness of the newly synthesized cartilage surface but also on chondrocyte proliferation and differentiation at the defect site; the main indicator of cartilage repair was the formation of layered chondrocytes in the proliferative tissue.

### 2.13. Statistical Analysis

All the data are presented as arithmetic means ± standard deviations (SD). All replicates were biological replicates using different samples of cryogel or different cell cultures. The statistical significance of differences between groups was determined by Student’s *t*-test with *p* < 0.05 indicating statistical significance, as follows: *p* < 0.05 (*); *p* < 0.01 (**); and *p* < 0.001 (***). Statistical analyses were performed with GraphPad Prism 8.0 (GraphPad Software, Inc., La Jolla, CA, USA).

## 3. Results

### 3.1. Water Content Analysis

**Figure 2:**
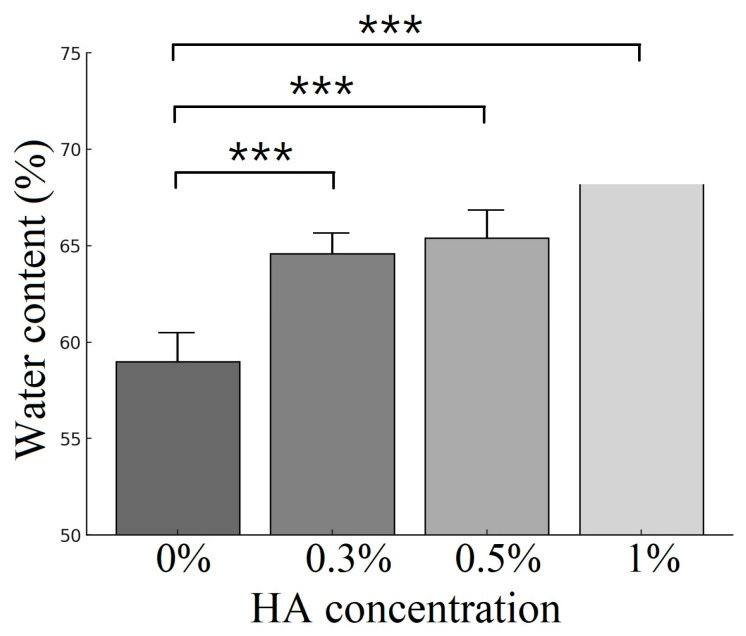
Water content of ECM-based cryogels with varying hyaluronic acid (HA) concentrations (data presented as mean ± SD, *n* = 10, *** *p* < 0.001).

To determine the optimum HA concentration of the ECM-based cryogel, we first aimed to determine the water content of the cryogels with different HA concentrations (0%, 0.3%, 0.5%, and 1%). The results indicate that the greater the HA concentration, the greater the water content of the cryogels when they were impregnated in ddH_2_O for 1 h (Figure 2). The three cryogels with different HA concentrations, including the cryogels with 0.3% HA (64.59 ± 1.79) (*p* < 0.0001), the lowest concentration of HA we tested; the cryogels with 0.5% HA (65.39 ± 2.01) (*p* < 0.0001); and the cryogels with the highest HA concentration of 1% (69.13 ± 1.11) (*p* < 0.0001), presented water content values significantly greater than that of the cryogel without HA (59.00 ± 1.97).

### 3.2. Swelling Ratio Analysis

**Figure 3:**
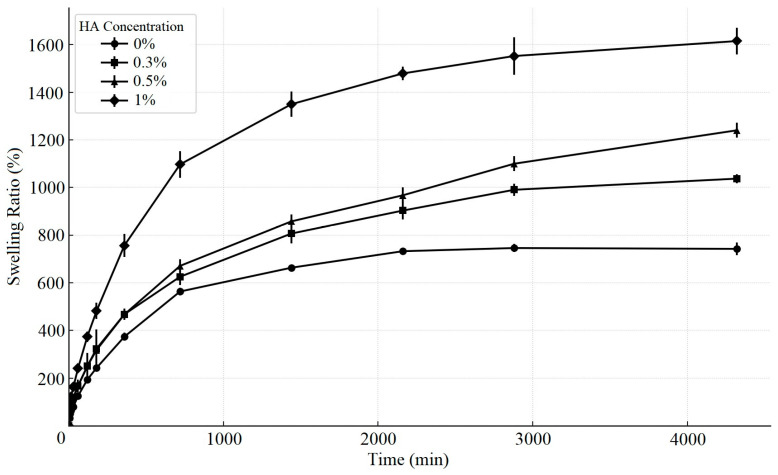
Swelling ratios of ECM-based cryogels with varying HA concentrations at different time points (data presented as mean ± SD, *n* = 10).

As the water content of the ECM-based cryogel impacts the swelling ratio of the material, we evaluated the swelling ratio of the cryogels with different HA concentrations. The results show that cryogels with higher HA concentrations exhibited greater maximum swelling ratios after impregnation with ddH_2_O, with the swelling ratios of the cryogels with all HA concentrations plateauing after 48 h (Figure 3). After 168 h, compared with cryogels without HA (742.3 ± 26.8), cryogels with different concentrations of HA presented significantly greater swelling ratios, including cryogels with 0.3% HA (1037.1 ± 19.0) (*p* < 0.0001), which increased to more than 10 times their initial weight, and cryogels with 0.5% HA (1240.2 ± 31.4) (*p* < 0.0001), with the highest maximum swelling ratio observed in cryogels with 1% HA (1614.9 ± 55.5) (*p* < 0.0001).

### 3.3. Porosity Analysis

**Figure 4:**
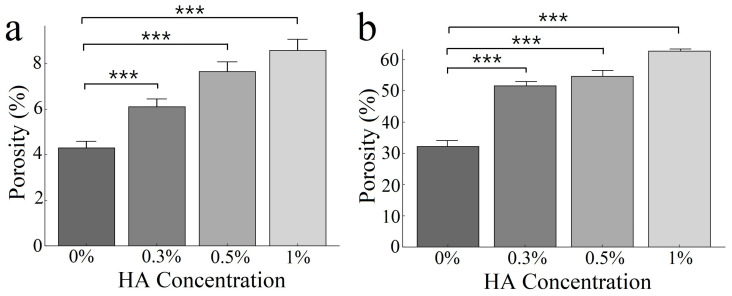
The porosity of ECM-based cryogels with varying HA concentrations. (**a**) Before swelling and (**b**) after swelling (data presented as mean ± SD, *n* = 10, *** *p* < 0.001).

The high swelling ratio of the ECM-based cryogels may correlate with the high porosity of the cryogels, which is critical for evaluating the effectiveness of the scaffold. The results indicate that the higher the concentration of HA in the cryogel, the greater the porosity in the cryogel both before (Figure 4a) and after swelling (Figure 4b) in water for 24 h, as measured using absolute ethanol. Compared with the cryogels without HA (4.30 ± 0.37), the cryogels with different concentrations of HA, including the cryogels with 0.3% HA (5.57 ± 0.42) (*p* < 0.0001), 0.5% HA (7.66 ± 0.62) (*p* < 0.0001), and 1% HA (8.58 ± 0.61) (*p* < 0.0001), presented significantly greater porosity before swelling. The same trend was observed for the cryogels after swelling, with the cryogels with 0.3% HA (52.00 ± 0.40) (*p* < 0.0001), 0.5% HA (56.80 ± 1.49) (*p* < 0.0001), and 1% HA (66.64 ± 2.91) (*p* < 0.0001) exhibiting significantly greater porosity than the cryogel without HA (32.44 ± 1.82). Furthermore, after swelling for 24 h, the porosity in all of the cryogels increased to approximately eight times their initial porosity, and the three cryogels with different HA concentrations exhibited a porosity that was more than 50%.

### 3.4. Scanning Electron Microscopy Analysis

**Figure 5:**
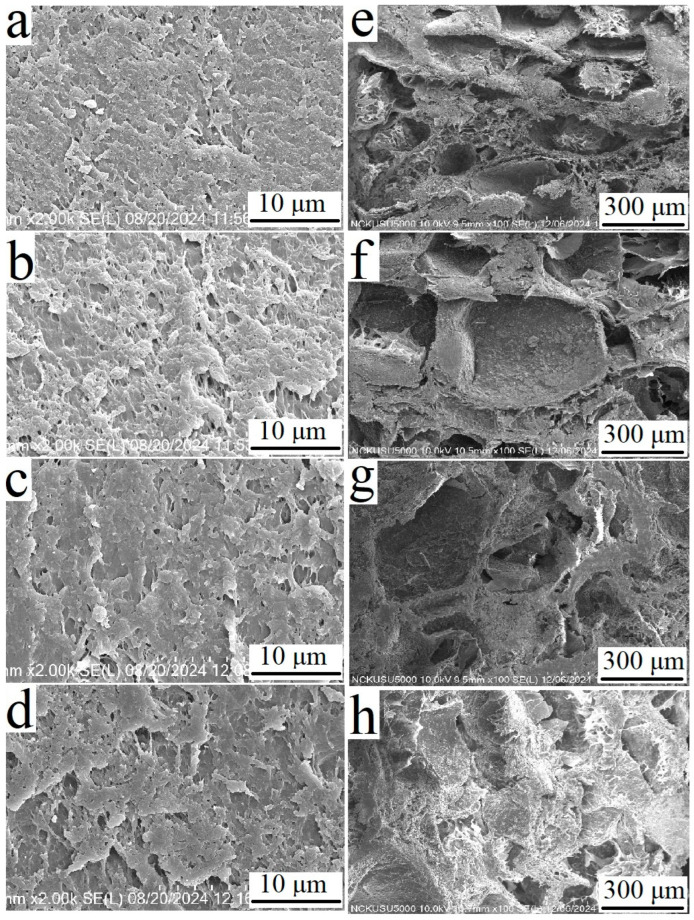
SEM micrographs of ECM-based cryogels with different HA concentrations. (**a**–**d**) Cryogels with 0% HA, 0.3% HA, 0.5% HA, and 1% HA before swelling, respectively; the scale bar represents 10 μm. (**e**–**g**) Cryogels with 0.3%, 0.5%, and 1% HA after swelling for 6 h, respectively; the scale bar represents 300 μm. (**h**) Bone marrow-derived mesenchymal stem cell (BM-MSC)-derived exosome-seeded cryogel with 0.3% HA after swelling for 6 h; the scale bar represents 300 μm.

As high porosities were found in the ECM-based cryogels with different HA concentrations, we aimed to further characterize the cryogels according to estimations of their pore sizes. SEM micrographs revealed that all of the cryogels had a similar appearance, with small pores that have diameters less than 5 μm before swelling, regardless of the HA concentration (Figure 5a–d). However, the pore sizes of the cryogels increased after impregnation in ddH_2_O for 24 h, with greater HA concentrations resulting in more distinct pores with larger diameters (Figure 5e–g). The pore diameter range for cryogels with each concentration of HA after swelling was 100–300 μm for the cryogels with 0.3% HA, 300–500 μm for the cryogels with 0.5% HA, and 350–600 μm for the cryogels with 1% HA. After BM-MSC-derived exosomes were seeded on cryogels with an HA concentration of 0.3%, the pores were still present (100–250 μm in diameter) (Figure 5h).

### 3.5. Mechanical Strength Analysis

**Figure 6:**
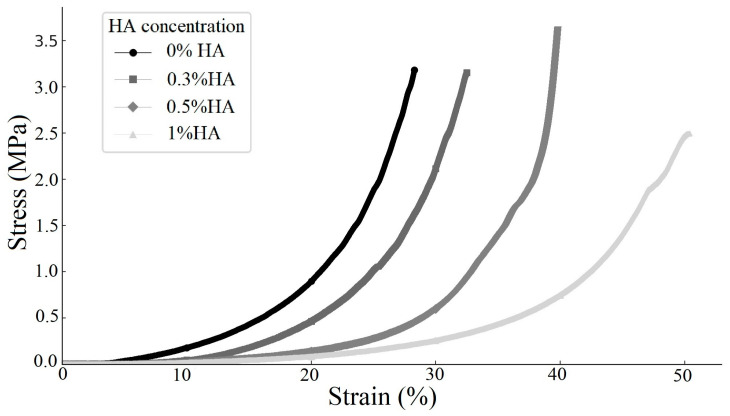
Stress–strain curves of ECM-based cryogels with various HA concentrations after swelling for 6 h (*n* = 3).

The ECM-based cryogel designed to assist in the repair of articular cartilage defects requires adequate mechanical strength to tolerate the compressional force during movement. The results revealed that, after impregnation in ddH_2_O for 6 h, the higher the HA concentration of the swollen cryogel, the shallower the slope on the linear section of the stress–strain curve, indicating a lower Young’s modulus and thus lower compressional stiffness of the material (Figure 6). The calculated Young’s modulus values for the cryogels with different concentrations of HA were 4.768 MPa for the cryogel with 0% HA, 3.381 MPa for the cryogel with 0.3% HA, 2.001 MPa for the cryogel with 0.5% HA, and 0.725 MPa for the cryogel with 1% HA.

### 3.6. Cell Viability Analysis

**Figure 7:**
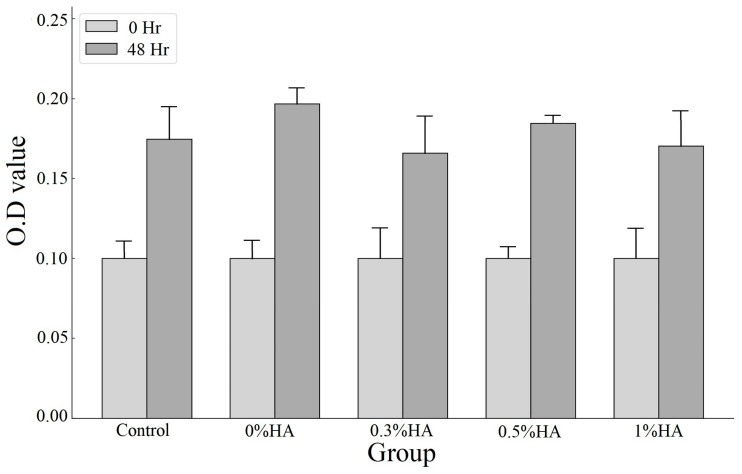
MTT assay of ECM-based cryogels with varying HA concentrations compared with the control (data presented as mean ± SD, *n* = 5).

The MTT assay with L929 cells indicated that, regardless of the concentration of HA in the ECM-based cryogel, the optical density of the cultured cells increased 48 h after cryogel implantation. No significant difference in the number of cultured cells at 48 h was observed after the implantation of cryogels with different concentrations of HA (0% HA: 0.200 ± 0.008, *p* = 0.137; 0.3% HA: 0.170 ± 0.020, *p* = 0.831; 0.5% HA: 0.189 ± 0.037, *p* = 0.564; 1% HA: 0.176 ± 0.031, *p* = 0.911) compared with the control condition with no cryogel implanted at 48 h (0.174 ± 0.023) (Figure 7). The results demonstrate that the materials had no cell toxicity.

As the ECM-based cryogel is a biomaterial scaffold designed to fill chondral defects, sufficient stress resistance is needed to sustain the high-pressure load of articular cartilage. Compared with the cryogels without HA, the cryogels with 0.3% HA had more favorable water content and swelling ratio values and adequate porosity and pore size values, which were significantly higher compared with cryogels with no HA, and Young’s modulus value was greater than those of cryogels with 0.5% and 1% HA. Therefore, we selected cryogels with 0.3% HA for further experiments, including both in vivo and in vitro studies.

**Figure 8:**
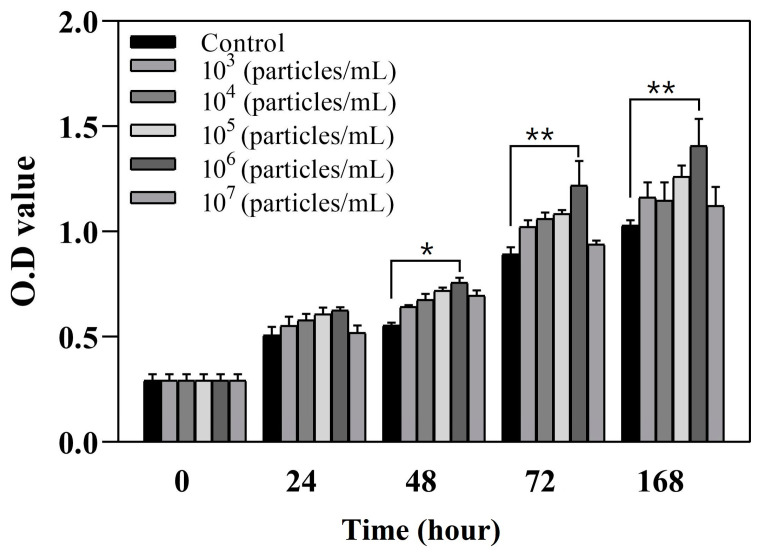
MTT assay of chondrocyte cultures with various concentrations of BM-MSC-derived exosomes compared with the control condition (data presented as mean ± SD, *n* = 5, * *p* < 0.05, ** *p* < 0.01).

An MTT assay revealed greater viability for chondrocytes cultured with different concentrations of BM-MSC-derived exosomes than for the control culture without the addition of exosomes after 168 h (1.029 ± 0.025), especially for the chondrocytes cultured with an exosome concentration of 10^6^ particles/mL (1.407 ± 0.128, *p* = 0.002), which had the highest cell viability (Figure 8). A higher concentration of exosomes resulted in a higher chondrocyte optical density, but the highest exosome concentration of 10^7^ particles/mL (1.122 ± 0.078, *p* = 0.063) did not lead to significantly greater cell growth compared with the control condition, indicating that excessively high concentrations may not be beneficial. Thus, we selected the highest exosome concentration that had a significant difference compared with the control condition for exosome seeding. As a result, we seeded BM-MSC-derived exosomes at a concentration of 10^6^ particles/mL onto 0.3% HA ECM-based cryogels for further in vivo and in vitro experiments.

### 3.7. Bone Marrow-Derived Mesenchymal Stem Cell-Derived Exosome Analysis

**Figure 9:**
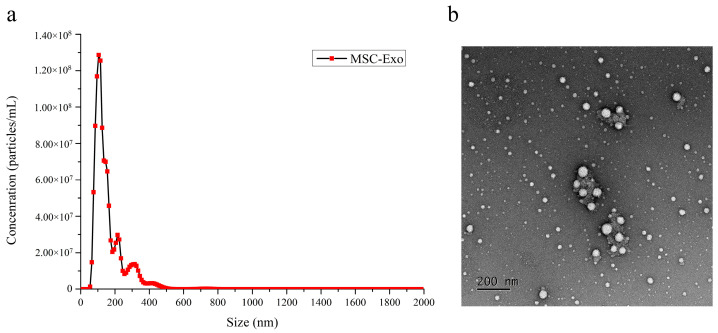
(**a**) Relative concentration, measured as particles/mL, of BM-MSC-derived exosomes of varying sizes; (**b**) TEM micrograph of BM-MSC-derived exosomes.

NTA of the BM-MSC-derived exosomes revealed a heterogeneous population of particles with sizes ranging from 50 to 500 nm; however, the majority of the population was 50–200 nm in size (Figure 9a). The TEM micrograph further supported the NTA results (Figure 9b). Thus, the data confirmed that we successfully isolated exosomes by centrifugation, supporting the feasibility of investigating the efficacy of using these exosomes in subsequent experiments.

### 3.8. Chondrocyte Proliferation

**Figure 10:**
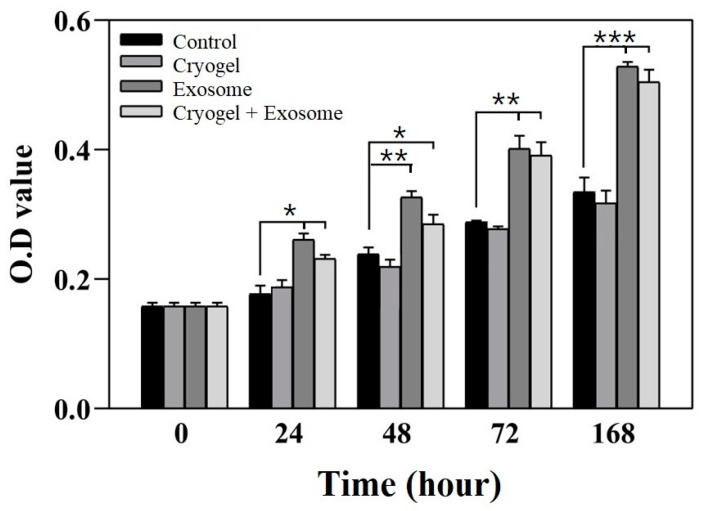
MTT assay of chondrocyte cultures after cryogel implantation, exosome addition, and exosome-seeded cryogel implantation compared with the control condition (data present as mean ± SD, *n* = 5, * *p* < 0.05, ** *p* < 0.01, *** *p* < 0.005).

Before conducting in vivo experiments, the ECM-based cryogel, BM-MSC-derived exosomes, and the exosome-seeded cryogel were tested for chondrocyte cytotoxicity and proliferation using an MTT assay. The MTT assay using extracted chondrocytes indicated that, compared with the control conditions, both the experimental condition with exosomes and the experimental condition with the exosome-seeded cryogel resulted in significantly greater cell optical density at all time intervals (Figure 10). At 168 h, the cell optical density values were approximately 58% and 51% greater for the experimental condition with exosomes (0.529 ± 0.006, *p* < 0.0001) and the exosome-seeded cryogel (0.505 ± 0.016, *p* < 0.0001), respectively, than for the control condition (0.335 ± 0.019), with no significant differences between the two experimental conditions. No significant difference was observed between the cryogel condition (0.328 ± 0.020, *p* = 0.623) and the control condition. These results demonstrate that the treatments presented no cytotoxicity, especially the exosome and the exosome-seeded cryogel treatments, both of which maintained cell viability and promoted chondrocyte proliferation.

**Figure 11:**
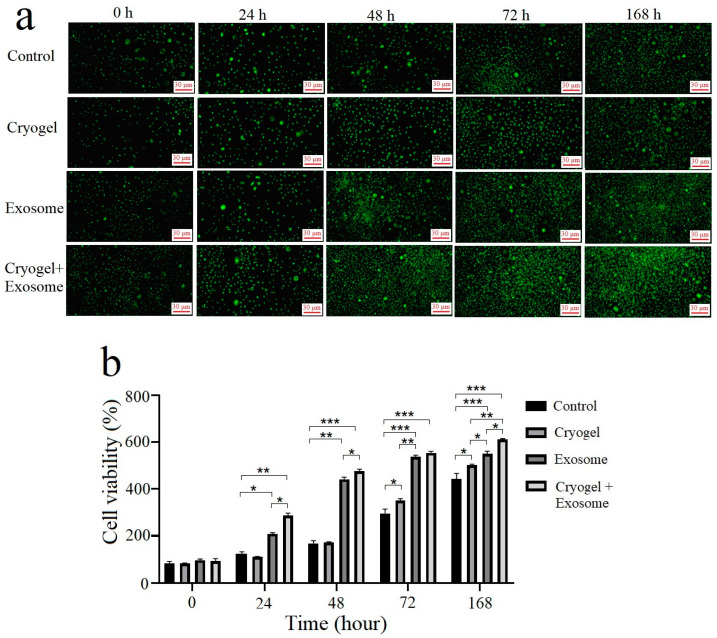
(**a**) Calcein AM staining of seven-day chondrocyte cultures after cryogel implantation, exosome addition, and exosome-seeded cryogel implantation compared with the control condition. Each scale bar represents 30 μm; (**b**) The cell viability analysis of Calcein AM staining was measured using Image (data presented as mean ± SD, *n* = 5, * *p* < 0.05, ** *p* < 0.01, *** *p* < 0.005).

The fluorescence images of Calcein AM-stained samples after cell culture for 168 h revealed that all conditions increased in fluorescence intensity over time, indicating chondrocyte proliferation (Figure 11). The exosome alone and exosome-seeded cryogel conditions significantly differed from the control condition at all time intervals. However, although the cryogel alone condition did not significantly differ from the control condition at 24 h and 48 h, it presented significantly greater intensity compared with the control condition at 72 h and 168 h. Furthermore, the exosome alone and exosome-seeded cryogel conditions still significantly differed from the cryogel alone condition at all time intervals. Specifically, at 168 h, compared with the control condition (442.04 ± 20.98), all the experimental conditions, including the cryogel alone (501.83 ± 3.98, *p* = 0.024), exosomes alone (550.81 ± 8.84, *p* < 0.0001), and the exosome-seeded cryogel (610.91 ± 3.66, *p* < 0.0001) displayed significantly greater fluorescence intensity, especially the exosome-seeded cryogel condition, indicating that more cells were present in the chondrocyte cultures and thus greater cell viability was achieved.

### 3.9. Biochemical Analysis

**Figure 12:**
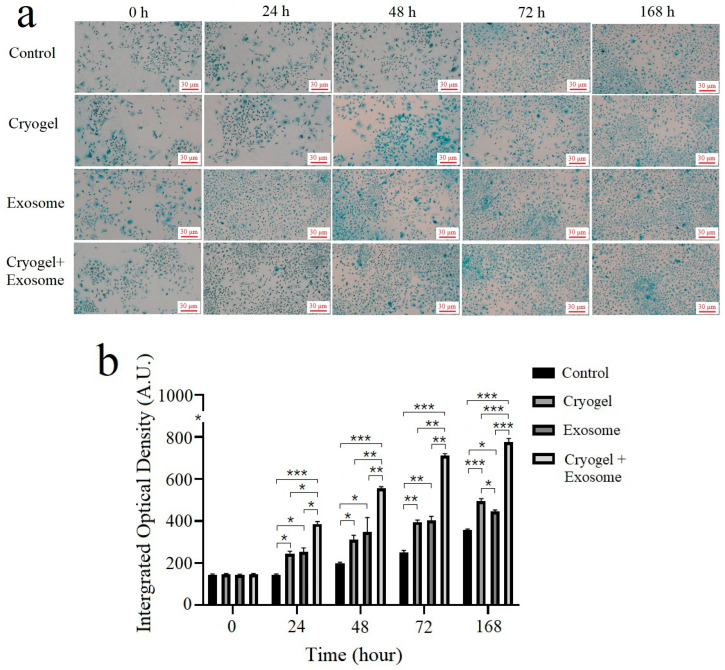
(**a**) Alcian blue staining of 168 h chondrocyte cultures after cryogel implantation, exosome addition, and exosome-seeded cryogel implantation compared with the control condition; each scale bar represents 30 μm. (**b**) The integrated optical density analysis of Alcian blue staining was measured using ImageJ (data presented as mean ± SD, *n* = 5, * *p* < 0.05, ** *p* < 0.01, *** *p* < 0.005).

Alcian blue staining was used to observe the ECM of chondrocyte cultures for 168 h. The staining results revealed that all experimental conditions significantly increased the Alcian blue staining intensity compared with control condition at all time intervals (Figure 12). However, at 168 h, both the cryogel alone (500.12 ± 9.94, *p* < 0.0001 and *p* = 0.0001) and exosome-seeded cryogel (775.71 ± 15.02, *p* < 0.0001 and *p* < 0.0001) conditions displayed significantly greater integrated optical density compared with the control (359.24 ± 4.20) and exosome alone (444.88 ± 7.25) conditions. Additionally, the exosome-seeded cryogel condition resulted in a greater ECM synthesis than the cryogel alone and exosome alone conditions at all time intervals significantly. Therefore, although all three experimental conditions significantly increased chondrocyte activity for ECM synthesis, the cryogel alone and exosome-seeded cryogel conditions demonstrated greater continual effect, up to 168 h, compared with the exosome alone condition.

**Figure 13:**
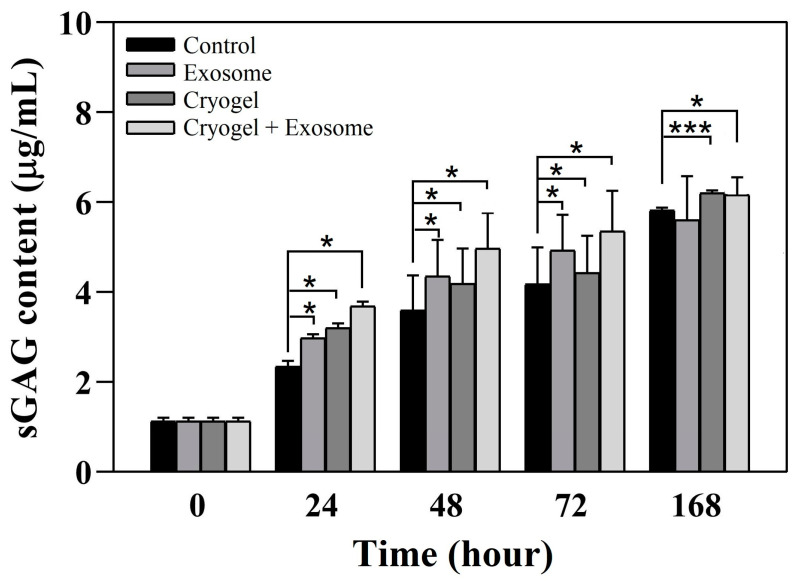
sGAG content of chondrocyte culture media after cryogel implantation, exosome addition, and exosome-seeded cryogel implantation compared with the control condition (data presented as mean ± SD, *n* = 5, * *p* < 0.05, *** *p* < 0.001).

To quantify the sGAG content of chondrocyte cultures treated with the ECM-based cryogel and BM-MSC-derived exosomes, we performed further biochemical analyses. The results show that, although all three experimental conditions resulted in significantly higher sGAG contents than did the control condition for up to 72 h, only the cryogel (6.204 ± 0.058, *p* = 0.0006) and the exosome-seeded cryogel conditions (6.159 ± 0.158, *p* = 0.036) result in significantly greater concentrations of sGAG at 168 h than did the control condition (5.932 ± 0.060) (Figure 13). Exosomes alone (5.606 ± 0.972, *p* = 0.528) did not significantly differ from the control condition at 168 h. These results indicate that the cryogel alone and the exosome-seeded cryogel continuously increase the amount of sGAG synthesized by chondrocytes. Therefore, these data suggest that the exosome-seeded cryogel promotes chondrocyte proliferation and ECM synthesis, both of which are important for articular cartilage defect repair.

### 3.10. In Vivo Implantation Analysis

**Figure 14:**
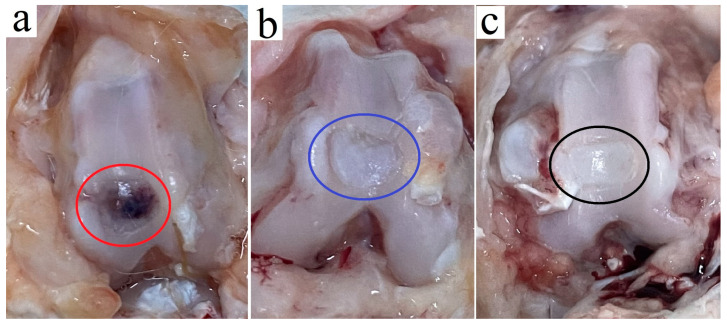
Gross evaluation of articular cartilage defects 4 weeks after the operation in a rabbit model. (**a**) Control group defect (red circle); (**b**) ECM-based cryogel group defect (blue circle); (**c**) BM-MSC exosome-seeded ECM-based cryogel group defect (black circle).

Furthermore, we performed an in vivo animal experiment to confirm the effectiveness of the BM-MSC-derived exosome-seeded ECM-based cryogel for articular cartilage defect repair. Defects were surgically induced in the femoral knee articular cartilage of rabbit models, with the limbs categorized into three groups. Four weeks after the operation, for the control group without the exosome-seeded cryogel, the chondral defect surface displayed no obvious chondral repair, with only a layer of connective tissue covering the defect (Figure 14a). In contrast, the cryogel group exhibited chondrocyte proliferation and cartilage regeneration at the defect site, but the newly synthesized cartilage surface was irregular and incomplete (Figure 14b). However, when the exosome-seeded cryogel was implanted in the initial defect, chondrocyte proliferation resulted in a regular and smooth articular cartilage surface with near-complete cartilage repair, which is the optimal condition after repair (Figure 14c).

### 3.11. Histological Analysis

**Figure 15:**
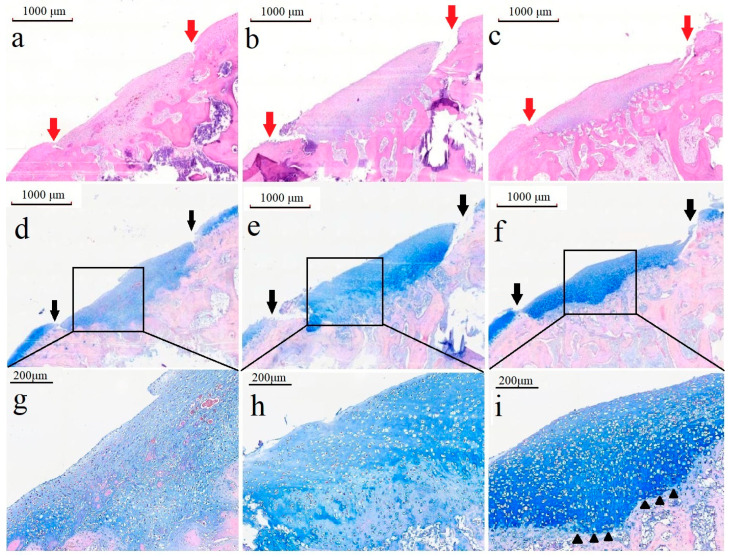
Histological images showing recovery of the cartilage defects after four weeks. H&E staining of defects in the control (**a**), ECM-based cryogel implantation (**b**), and exosome-seeded cryogel implantation (**c**) groups. Red arrows in (**a**–**c**) indicate the defect margin. Low-magnification images of Alcian blue staining of defects in the control (**d**), ECM-based cryogel implantation (**e**), and exosome-seeded cryogel implantation (**f**) groups. Black arrows in (**d**–**f**) indicate the defect margin. High-magnification images of Alcian blue staining in the control (**g**), ECM-based cryogel implantation (**h**), and exosome-seeded cryogel implantation (**i**) groups. The tidemark (black triangles) was clearly observed.

We further characterized the articular cartilage defect site via H&E and Alcian blue staining of longitudinal sections. For the H&E staining of the control group, a layer of fibrous tissue with an irregular surface filled the defect. The ECM-based cryogel group had a thickened layer over the defect with an irregular gap around the peripheral edge, whereas the BM-MSC-derived exosome-seeded cryogel group had a smoother proliferative tissue layer in the defect (Figure 15a–c).

For the Alcian blue staining, the articular cartilage defect in the control group had less ECM and fewer chondrocytes with an irregular arrangement, whereas the ECM-based cryogel group had a thickened cartilage layer with more chondrocyte growth and good ECM proliferation; however, a normal arrangement of the cartilage layers was not observed, and the tidemark was not clearly observed. The BM-MSC-derived exosome-seeded cryogel group proved to be the most effective, with the highest chondrocyte proliferation and a normal arrangement of chondrocytes in the defect, as demonstrated by the different orientations of the chondrocytes; the tidemark was also clearly observed (Figure 15d–i).

## 4. Discussion

Our study demonstrated that the BM-MSC-derived exosome-seeded ECM-based cryogel promotes chondrocyte proliferation and ECM synthesis, displaying potential for the repair of articular cartilage defects. Water molecules, which can be absorbed from blood plasma and can diffuse into the cryogel scaffold, are essential for cell metabolism [31] and therefore contribute to chondrocyte growth, proliferation, and survival, consistent with the goal of biocompatibility. In addition, the hydrophilic material of HA has an adequate swelling ratio due to its high negative charge, enabling the cryogel to expand by binding to water molecules [32] and assisting in the repair process by fully filling the defect. A concentration of HA that promotes a high water content and swelling ratio would therefore be able to increase the proliferation of cells and the regeneration of articular cartilage, as the water potential inside the ECM-based cryogel could be equilibrated with the external ECM environment [33].

A high swelling ratio denotes high porosity for cryogels [29], and high interconnected porosity and large pore diameter promote nutrient delivery and cell migration into the scaffold [34]. Previous research has indicated that cryogel scaffolds with pore sizes of 100–200 µm assist in cartilage repair by promoting significantly greater chondrocyte proliferation, chondrogenic differentiation, and cartilage-like ECM deposition. In comparison, scaffolds with smaller pore sizes, such as 50 µm, have the disadvantage that oxygen and nutrients cannot be transported easily, and scaffolds with larger pore sizes, such as 400 µm, provide a larger environment but increase the time needed for cells to fill the pores [35]. Pores with diameters between 150 and 250 μm promote cartilage regeneration, with the greater gene expression and production of aggrecan and collagen type II, enabling cell–cell interactions and facilitating the filling of the defect by chondrocytes [36]. Our results demonstrate that the ECM-based cryogels with 0.3% HA displayed pore sizes of 100–300 μm after swelling, which is comparable with the optimal range for cartilage repair. Our 0.3% HA ECM-based cryogel, intended to promote the repair of articular cartilage, displays a high porosity of 52% after swelling, comparable to that of human cartilaginous joints, which have a porosity range of 30–95% [37]. As a result, our ECM-based cryogel provides a suitable environment for chondrocyte proliferation and articular cartilage defect repair.

Although a scaffold with high porosity facilitates cell proliferation and ECM deposition, the mechanical properties of the scaffold should also resemble those of native cartilage for it to be deemed suitable for cartilage repair [38]. Sufficient mechanical strength of the cryogel scaffold is imperative for developing biomaterials that achieve good performance under load because it prevents product disintegration [39]. We selected cryogel as our material because the fracture stress of cryogels is 70 times greater than that of hydrogels, which in turn is because cryogelation and polymerization result in the formation of a dense polymer with thick pore walls [40]. We selected an HA concentration of 0.3% for our material, which has Young’s modulus of 3.381 MPa; this value is greater than that of human articular cartilage (1.03 ± 0.48 MPa) [41], indicating that the cryogel is sufficiently strong to support articular cartilage physiological compression. Our ECM-based cryogels have favorable porosity, pore diameter, swelling potential, and mechanical properties, indicating that they are optimal for tribological articular cartilage regeneration.

The majority of our population of BM-MSC-derived exosomes had sizes of 50–200 nm, similar to those reported in previous studies, with a similar size distribution curve and a range of 30–200 nm, with most exosomes having a diameter of 150 nm [42]. Our biomaterials with all HA concentrations demonstrated no cytotoxicity, indicating that they are suitable for in vivo implantation and do not influence crucial cell maintenance processes such as cell survival, cell proliferation, and biochemistry. Although numerous studies on exosomes have shown promising results regarding the ability to promote cartilage repair, no in vivo studies have been conducted to determine the optimal concentration of exosomes [43]; our results indicate that BM-MSC-derived exosomes at a concentration of 10^6^ particles/mL promote increased chondral cell growth after 168 h, whereas the addition of exosomes at a concentration of 10^7^ particles/mL do not significantly improve chondrocyte proliferation. Cellular stress from an overload of biomolecules may impair specific cellular functions, such as proliferation or differentiation, as a result of increased metabolic stress on target cells and disruption of normal functions.

In our MTT assay and Calcein AM staining, the exosomes alone and exosome-seeded cryogel conditions significantly increased chondrocyte proliferation. This finding aligns with previous exosome research, as MSC-derived exosomes have been proven to deliver essential molecules and information [44], facilitate the deposition of proteoglycans and collagen type II [45], and regulate chondrocyte apoptosis [24], enhancing the regeneration ability of cartilage.

In our study, after in vitro Alcian blue staining of proteoglycans in the ECM, a significant increase in intensity was observed for the cryogel alone and exosome-seeded cryogel conditions compared with the exosome alone condition at 168 h, which was also observed for the sGAG content analysis. We observed a significant increase in sGAG content for the exosome alone condition compared with the control condition for up to 72 h, but not at 168 h. In the exosome alone condition, ECM proliferation does not appear to be as continuous as in the cryogel alone or exosome-seeded cryogel conditions. This may be due to the decomposition of the exosomes over time. Some factors, such as the rapid clearance of exosomes by macrophages [46] and the tendency of exosomes to be removed via the circulatory system and accumulate in the spleen and liver [47], necessitate a higher exosome dose [48].

Another method is seeding exosomes in 3D biomaterial scaffolds such as cryogels [25] and hydrogels [49] to mimic the ECM environment of native articular cartilage while maintaining the stability and bioactivity of the exosomes and releasing exosomes in a controlled manner [50]. Cryogels can be used as working platforms to control the local release of exosomes by increasing the stability of proteins and mRNAs [51]. The cross-linking of the polymers creates a rigid structure that degrades gradually, enabling prolonged therapeutic effects in articular cartilage defects [52]. The release of exosomes results from diffusion, polymer dissolution or degradation, and swelling of the 3D scaffold [53]. Referring to our 72 h release profile analysis (Figure S2), approximately 90% of the seeded exosomes were released from the cryogel in the first 72 h, comparable to previous research [54]. An initial burst release was also observed, attributable to the high porosity and pore size of the ECM-based cryogel [29]. This release kinetic is consistent with previously reported trends in the literature, supporting the reliability of our cryogel scaffold in enabling rapid, yet continued, exosome delivery. Hence, our research revealed that the use of the ECM-based cryogel, seeded with exosomes, successfully extended the effects of the exosomes up to 168 h.

Our sGAG content analysis demonstrated that the cryogel alone and the exosome-seeded cryogel significantly facilitated ECM deposition by chondrocytes. The components of our ECM-based cryogels are gelatin, CS, and HA, all of which provide chondral tissue with a suitable 3D scaffold environment for ECM synthesis. In contrast, regarding chondrocyte proliferation, the cryogel alone affected cell proliferation less significantly than the exosomes alone. Previous studies have shown that exosome-mediated cartilage repair involves rapid cellular proliferation and infiltration due to exosomal CD73-mediated adenosine activation of AKT and ERK signaling [55]. Thus, exosomes play a key role during cell proliferation for articular cartilage defect repair.

The ECM-based cryogel assisted in cartilage repair and growth in vivo, with the defect filled with a uniform surface layer of articular cartilage with more chondrocytes. We observed not only increased sGAG content but also chondrocyte proliferation, indicating the effectiveness of the cryogel scaffold in assisting chondrocyte growth. More chondral proliferation and an appropriate chondrocyte arrangement were observed in defects treated with exosome-seeded cryogels, indicating good differentiation and physiological repair attributable to the exosomes. The tidemark was also clearly observed, delineating the boundary between uncalcified cartilage and calcified cartilage within the articular cartilage. Its presence and proper formation are critical for evaluating the functional integration and biomechanical stability of repaired cartilage [56]. Therefore, the results demonstrate that BM-MSC-derived exosomes aid in differentiation and result in layered chondrocyte arrangement during articular cartilage defect repair.

No signs of immune or inflammatory response were observed at the implantation site for all groups in our in vivo experiment. Cryogels have been shown to be immunocompatible and lack immunostimulatory activity, making them minimally invasive materials possible for utilization in medical fields [57]. Furthermore, the use of exosomes in cartilage repair can modulate inflammation and minimize immune rejection [26]. Thus, our experiment correlates with previous studies, highlighting that cryogels and exosomes are promising for articular cartilage regeneration.

Previous research has yielded similar results. An in vivo study of rabbits showed that CGC cryogels resulted in increased accumulation of homogenous cartilage ECM after 8 weeks of surgery [58]; however, the study evaluated the effect of cryogel alone, without exosome seeding. Further in vivo studies also reported that BM-MSC-derived exosomes attenuate cartilage damage in the context of osteoarthritis and restore cartilage homeostasis by exerting a protective effect against cartilage degradation [25,59]; nonetheless, the studies tested the effect of exosomes alone, without seeding them in cryogel. In our in vivo and in vitro study, we utilized the combination of the cryogel and exosomes for articular cartilage defect repair. No previous studies have investigated the combination of cryogels containing HA and exosomes in vivo; only one in vitro study has demonstrated an increase in cell proliferation after treatment with articular chondrocyte-derived exosomes combined with CGC cryogel extract for articular cartilage repair [29]. Our study provides evidence in vivo that the implantation of BM-MSC-derived exosome-seeded ECM-based cryogels significantly promotes cartilage repair.

However, our study still presents some limitations. First, as our in vivo experiment only lasted four weeks, it may not be sufficient to assess the long-term durability and integration of the exosome-seeded cryogels in cartilage repair. However, our study is a preliminary study with a low sample size and short duration designed to confirm the efficacy of the exosome-seeded cryogel; future research with a larger sample size can be conducted to assess the long-term outcome and ensure statistical power. Additionally, a single batch of BM-MSC exosomes limits the ability of this preliminary study to determine the effects of batch-to-batch variability on the efficacy of the exosome-seeded cryogels; future experiments using different batches of exosomes can be considered to further validate the result. Lastly, the in vitro and in vivo experiments were carried out with rabbit chondrocytes and rabbit models, which may not fully replicate human joint physiology and the complex mechanical loading environment of human articular cartilage.

## 5. Conclusions

This study characterized BM-MSC-derived exosome (at a concentration of 10^6^ particles/mL)-seeded 0.3% HA ECM-based cryogels and demonstrated their effectiveness in facilitating articular cartilage defect repair through an in vivo experiment in a rabbit model. The cryogels with increasing concentrations of HA presented increased water content, swelling ratio, and porosity values, both before and after swelling, and increased pore sizes after swelling but reduced Young’s modulus. We determined that an ECM-based cryogel containing 0.3% HA and a BM-MSC-derived exosome concentration of 10^6^ particles/mL were the optimal conditions for articular cartilage repair without cell cytotoxicity. Both the exosomes alone and the exosome-seeded cryogel significantly increased chondrocyte proliferation, assessed via an MTT assay and Calcein AM staining. Both the cryogel alone and the exosome-seeded cryogel significantly increased the sGAG content of chondrocyte cell cultures, with greater ECM synthesis, assessed via Alcian blue staining. Furthermore, our in vivo experiment results indicate that exosomes assist in the formation of layered chondrocytes, resulting from proper cell differentiation and chondrocyte proliferation. In conclusion, BM-MSC-derived exosome-seeded ECM-based cryogels have the potential for articular cartilage repair.

## Data Availability

The raw data supporting the conclusions of this article will be made available by the authors on request.

## Supplementary Materials

The following supporting information can be downloaded at https://www.mdpi.com/article/10.3390/polym17070975/s1, Figure S1: Exosome encapsulation analysis of BM-MSC exosomes seeded in ECM-based cryogel (data presented as mean ± SD, *n* = 5); Figure S2: Release profile analysis of BM-MSC exosomes seeded in ECM-based cryogel (data presented as mean ± SD, *n* = 5); Figure S3: Histological images of H&E and Alcian blue staining showing recovery of the cartilage defects of the second replicate after four weeks.
