# Supplementary material for: Exosome-Seeded Cryogel Scaffolds for Extracellular Matrix Regeneration in the Repair of Articular Cartilage Defects: An In Vitro and In Vivo Rabbit Model Study
Source: Polymers (Basel). 2025 Apr 3;17(7):975. doi: 10.3390/polym17070975 (PMC11991529; doi:10.3390/polym17070975)
Supplement: Supplementary file 1 [file polymers-17-00975-s001.zip › polymers-3547578-supplementary.pdf]

Supplementary Materials

for

Exosome-Seeded Cryogel Scaffolds for Extracellular Matrix Regeneration in the Repair of Articular Cartilage Defects: An In Vitro and In Vivo Rabbit Model Study

Daniel Yang [1,2], Joseph Yang [1,2], Shwu-Jen Chang [1,3], Jhe-Lun Hu [1,3], Yong-Ji Chen [1,3] and Shan-Wei Yang [4,5,6,*]

[1] Laboratory of Regenerative Medicine and Biosensors, I-Shou University, Kaohsiung City 824005, Taiwan; danielyang0930@gmail.com (D.Y.); josephyang0218@gmail.com (J.Y.); sjchang@isu.edu.tw (S.-J.C.); alanhu0812@gmail.com (J.-L.H.); ygchen2021@gmail.com (Y.-J.C.)

[2] Cambridge International Programme, St. Dominic Catholic High School, Kaohsiung City 802306, Taiwan

[3] Department of Biomedical Engineering, I-Shou University, Kaohsiung City 824005, Taiwan

[4] Department of Orthopedics, Kaohsiung Veterans General Hospital, Kaohsiung City 813414, Taiwan

[5] School of Nursing, Fooyin University, Kaohsiung City 831301, Taiwan

[6] Department of Leisure and Sports Management, Cheng Shiu University, Kaohsiung City 833301, Taiwan

* Correspondence: swyang3028@gmail.com; Tel.: +886-7-342-2121 (ext. 73048)

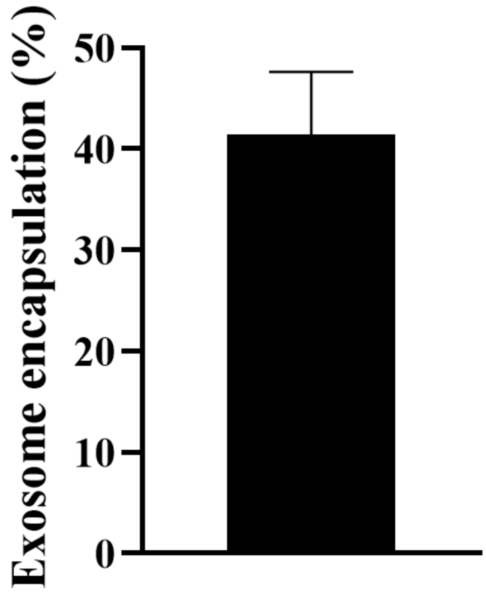

Figure S1. Exosome encapsulation analysis of BM-MSC exosomes seeded in ECM-based cryogel (data presented as mean ± SD, $n = 5$).

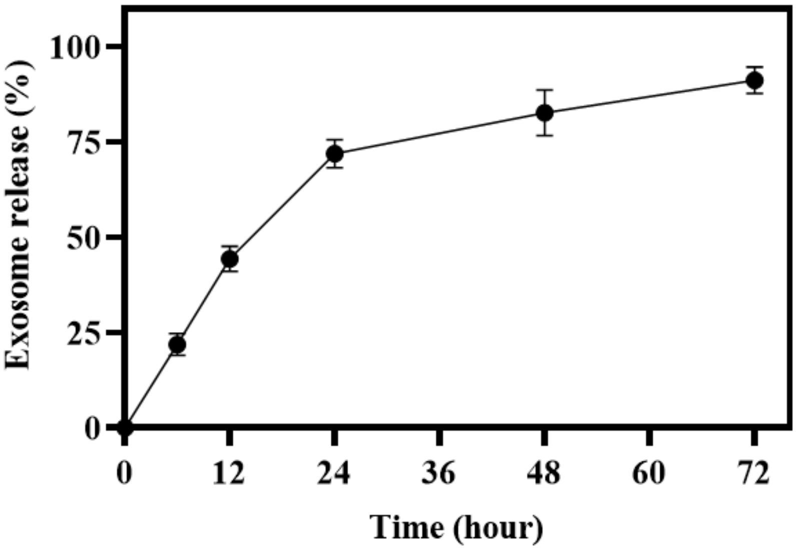

Figure S2. Release profile analysis of BM-MSC exosomes seeded in ECM-based cryogel (data presented as mean ± SD, $n = 5$).

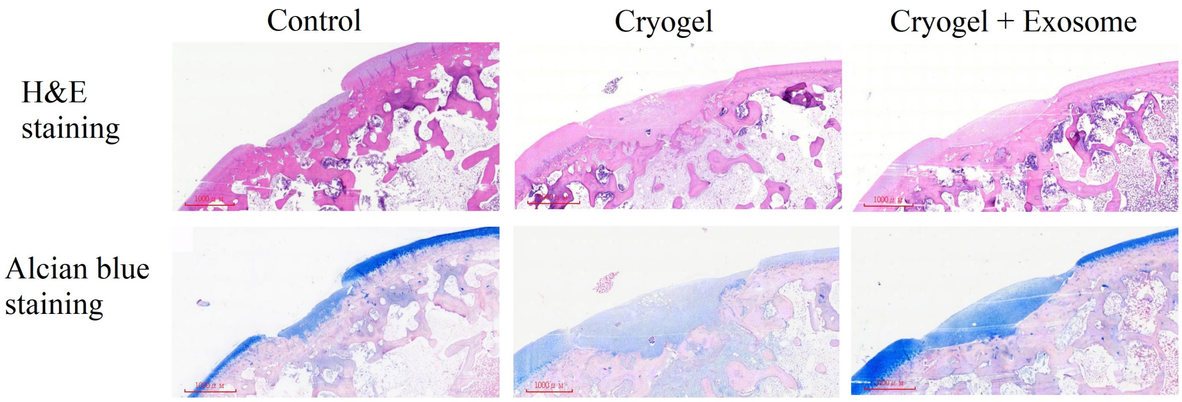

Figure S3. Histological images of H&E and Alcian blue staining showing recovery of the cartilage defects of the second replicate after four weeks.
